# Maximizing the potential of EEG as a developmental neuroscience tool

**DOI:** 10.1016/j.dcn.2023.101201

**Published:** 2023-01-27

**Authors:** George A. Buzzell, Santiago Morales, Emilio A. Valadez, Sabine Hunnius, Nathan A. Fox

**Affiliations:** a Department of Psychology, Florida International University, USA; b Center for Children and Families, Florida International University, USA; c Department of Psychology, University of Southern California, USA; d Department of Human Development and Quantitative Methodology, University of Maryland – College Park, USA; e Donders Institute for Brain, Cognition, and Behaviour, Radboud University, Nijmegen, the Netherlands

## 1 Introduction

Electroencephalography (EEG) is a relatively low-cost and non-invasive method for directly measuring brain activity, well-suited for capturing real-time neural information across the lifespan. EEG has been central to the discovery of fundamental developmental phenomena (e.g., Cellier et al., 2021; Marshall et al., 2002; Uhlhaas et al., 2010), and holds tremendous potential for further advancing research on child development. As a developmental neuroscience tool, the power of EEG lies in the analysis approaches used to extract meaningful information from the raw EEG signal. However, increasing analytic complexity has created substantial knowledge barriers that must be overcome before these approaches can be widely utilized by the field. This special issue in Developmental Cognitive Neuroscience presents a series of papers–with accompanying tutorials–focused on EEG analysis approaches that have not yet been widely adopted by developmental scientists. Geared toward novice and experienced researchers alike, all articles in this issue not only explain the theoretical and conceptual steps involved in each approach, but each article is accompanied by a step-by-step tutorial, publicly available code, and example data. Consistent with similar initiatives by other groups (Clayson et al., 2022, Kujawa and Brooker, 2022, Weisz and Keil, 2022) and the broader movement towards open-science (Foster and Deardorff, 2017, Markiewicz et al., 2021, Molloy, 2011, Munafò et al., 2017), it is our hope that improved access to methodological resources will further accelerate the pace of discovery within the field of developmental cognitive neuroscience.

## 2 Unique benefits of EEG as a developmental neuroscience tool

EEG reflects a rich source of neural information, providing direct, real-time measurement of brain activity. Specifically, EEG measures time-varying changes in the brain’s electric field, which reflects the summation of local extracellular fields, driven by transmembrane currents (Buzsáki et al., 2012, Nunez and Srinivasan, 2006). The fact that EEG is a direct measure of brain activity has several implications, including high temporal resolution of the EEG signal (Luck, 2014). Such information is commonly leveraged in the event-related potential (ERP) technique, to provide a temporal “marker” of cognitive events (Luck, 2014). However, as a direct measure of electrical activity, this also allows for quantifying power and phase dynamics of the brain’s electric field (Cohen, 2014). Power and phase information not only yield additional markers of cognitive function (Cavanagh and Frank, 2014, Klimesch, 2012), but may reflect the direct readout of neural mechanisms that guide information processing in the brain (Buzsaki, 2004, Fries, 2005, Herrmann et al., 2016, Singer, 1999) and which govern developmental phenomena (Cellier et al., 2021, Marshall et al., 2002, Uhlhaas et al., 2010). Further, such analyses of power and phase can be performed across temporal and spatial scales (Cohen, 2014), revealing unique insight into how particular neural dynamics map onto cognitive function(s) and/or change across development. EEG can also serve as a conceptual bridge linking the results of non-invasive EEG studies and results obtained from invasive techniques that also measure the brain’s electric field, such as local field potential (LFP) recordings and electrocorticography (ECOG) (Buzsáki et al., 2012, Narayanan et al., 2013). More broadly, the fact that EEG is a direct measure of the brain’s electric field opens the door for countless other analytical approaches, including quantifying the ratio of excitatory and inhibitory activity (Ostlund et al., 2022), localizing electrical generators (e.g., source localization approaches, Conte and Richards, 2022, Xie et al., 2022), quantifying information content (e.g., Ashton et al., 2022; Ng et al., 2022, Puglia et al., 2022), or performing model-based analyses to reveal dynamic statistical relations between brain and behavior (e.g., Jessen et al., 2021).

## 3 Importance of sharing developmental EEG methods

While the nature of the EEG signal affords countless opportunities to extract meaningful information about neural function, to date, the majority of developmental EEG studies have leveraged only a small subset of possible analytic approaches. Morales and Bowers (2022) note that, as of June of 2021, ∼ 77 % of developmental EEG studies published in *Developmental Cognitive Neuroscience* employ the ERP technique, ∼ 15 % utilize Fourier-based analyses of power, and the remaining 8 % account for all other analytic approaches (including time-frequency analyses). One reason for the limited use of other EEG analytic approaches may be that researchers simply lack relevant examples of how to employ such approaches in their own work. In line with the move towards open science (Foster and Deardorff, 2017, Markiewicz et al., 2021, Molloy, 2011, Munafò et al., 2017), it is important that researchers share both their data and (well-commented) code, in order to facilitate adoption by other researchers (alongside other benefits of sharing code, including enhanced reproducibility and transparency). Moreover, when researchers leverage open-source tools, as opposed to proprietary software, and adopt standardized data formats (e.g., the Brain Imaging Data Structure; BIDS) (Gorgolewski et al., 2016; Meyer et al., 2021; Pernet et al., 2019), this further enhances the likelihood of widespread adoption by the field. However, in addition to normalizing the sharing of one’s data and code for all published studies, there is still a need to disseminate knowledge regarding complex analytical methods in ways that are accessible to novice researchers (i.e., through tutorials). Toward these ends, this special issue presents a series of tutorials, each dedicated to an analytical method not yet in wide use within the field of developmental EEG. Below, we provide an overview of the tutorials available in this issue.

## 4 Overview of articles in this special issue

EEG studies require several preprocessing steps to remove artifacts contained in the EEG signal (e.g., environmental noise, blinks, and muscle activity). This is especially true of developmental EEG data, which typically involve shorter recordings and are more prone to artifacts (Bell and Cuevas, 2012, Debnath et al., 2020, Gabard-Durnam et al., 2018, Leach et al., 2020). Despite the need for preprocessing, the specific steps involved in data cleaning vary significantly between research traditions and laboratories. Moreover, some preprocessing steps can involve subjective inputs by the researcher, creating a challenge for reproducibility and scalability of developmental EEG studies. In recent years, several automated preprocessing pipelines have been proposed, specifically designed for developmental EEG data (e.g., HAPPE and MADE) (Debnath et al., 2020, Gabard-Durnam et al., 2018). When applied to developmental EEG data, these pipelines have been shown to yield increased trial retention, compared to other pipelines designed for adults or using a traditional approach of simply conducting artifact rejection (Debnath et al., 2020, Debnath et al., 2020, Gabard-Durnam et al., 2018). Several studies in the special issue extend this body of methodological work by proposing specific preprocessing steps for unique developmental populations (e.g., newborns or infants) or improving on previously developed pipelines. For example, Monachino et al. (2022) adapt the original HAPPE pipeline to optimize it for also preprocessing data for ERP analyses. Kumaravel et al. (2022) propose the Newborn EEG Artifact Removal (NEAR) pipeline to remove artifacts in EEG recordings from human newborns. Fló et al. (2022) propose the Automated Pipeline for Infants Continuous EEG (APICE), which uses several artifact correction algorithms and adaptive thresholds for artifact detection. Importantly, these three novel pipelines are compared with existing pipelines (e.g., MADE), highlighting their added benefit. Finally, Puglia et al. (2022) developed a standardized pipeline to preprocess and estimate reliable measures of signal variability (multiscale entropy; MSE) which are traditionally removed from EEG analyses as noise.

The ERP technique (Luck, 2014) can provide a useful marker of cognitive processes–with high temporal resolution–and has dominated developmental EEG research for decades (Morales and Bowers, 2022). Nonetheless, there remains untapped value and potential for bias when ERPs are computed using traditional analytic techniques. Traditional ERP analyses, which generally involve averaging across trials and are quantified using raw scalp voltages, are prone to several limitations, including: 1) potential bias due to the exclusion of participants with too few artifact-free trials (particularly problematic for studies involving younger populations) (Heise et al., 2022); 2) limited spatial specificity due to volume conduction (Kayser and Tenke, 2015); and 3) superposition of neural components that may overlap in both time and space (Donchin, 1966). However, there are approaches available that can increase the utility of ERP research by addressing these key limitations. For example, Heise et al. (2022) provide an introduction to the use of linear mixed effects modeling of trial-level ERP data. They demonstrate how this approach yields more accurate and less biased results compared to the more traditional ERP approach, even when applied to participants with low trial counts. Conte and Richards (2022) present a pipeline for the source reconstruction of scalp-recorded ERPs using individualized, MRI-constrained head models. Their pipeline allows for employing either a participant’s own MRI or the most closely matching MRI from a freely available MRI database, based on the participant’s head measurements. Scharf et al. (2022) provide an introduction to principal components analysis (PCA) for developmental ERP data, highlighting its utility in disentangling the multiple underlying components for a given scalp-recorded ERP.

Several manuscripts in the special issue provide an introduction to methods that leverage power and phase information inherent in EEG signals. Morales and Bowers (2022) provide an accessible introduction to time-frequency analyses of EEG to quantify measures of signal strength (power), as well as signal consistency (phase) across trials and channels (i.e., to estimate measures of “connectivity”). Morales and Bowers also outline important future directions and limitations of traditional time-frequency analyses, such as distinguishing between transient bursts and sustained oscillations, as well as defining widows of interest across time and frequency. Other articles in the special issue address these issues. For example, Rayson et al. (2022) provide a tutorial for techniques that can distinguish between rhythmic (sustained) versus transient activity within a specific frequency band. As an example of how to apply this technique, they focus on the analysis of beta bursts in infant EEG data. Buzzell et al. (2022) demonstrate the utility of applying PCA to time-frequency data, as both a data-reduction tool and a means to identify separate, meaningfully-distinct components of interest. Xie et al. (2022) introduce two pipelines to facilitate time-frequency functional connectivity analyses in cortical source space, improving the spatial specificity of such analyses. Sommer et al. (2022) demonstrate the application of multivariate neural pattern similarity analysis to time-frequency data, which allows for assessing the information content embedded within time-frequency phenomena, as opposed to only examining raw increases or decreases in activity levels. Figueira et al. (2022) present a set of tools to facilitate the analysis of steady-state visual evoked potentials (ssVEPs) in frequency tagging studies. Finally, Ostlund et al. (2022) provide a tutorial on how to parameterize the EEG power spectra, leveraging a recently developed algorithm (Donoghue et al., 2020) that allows for more appropriate interpretation of the physiological mechanisms underlying periodic and aperiodic EEG activity.

Articles in this special issue also demonstrate current approaches for quantifying statistical patterns within EEG data, as well as methods for improving statistical rigor and reproducibility. Traditional hypothesis testing in EEG research typically focuses on whether a restricted set of channel(s) and time point(s) differs across one or more conditions. However, such tightly confined analyses are likely to miss important, but perhaps unexpected effects at other time points or channels. This is especially problematic given the distributed nature of neural activity across multiple brain regions and networks (Lynn and Bassett, 2019). Similarly, conditions may differ in more complicated patterns of distributed activity that are masked by only comparing average activity levels. Ng et al. (2022) and Ashton et al. (2022) address this limitation. They present frameworks for applying machine learning techniques to infant task-based EEG data for the classification of brain states elicited by distinct stimuli, incorporating data from across the entire scalp and across the entire epoch. Moreover, Jessen et al. (2021) demonstrate how EEG analyses can be taken outside the confines of typical cognitive neuroscience tasks to more ecologically valid paradigms. Their tutorial illustrates how linear models, in conjunction with dynamic social stimuli (e.g., a recording of a mother reading a children’s story), can be used to characterize infants’ social processing in a naturalistic task. Finally, Meyer et al. (2021) present methods for organizing, analyzing, and reporting developmental EEG data to improve reproducibility and replicability. They provide an introduction to organizing data with the BIDS (Gorgolewski et al., 2016, Pernet et al., 2019), as well as demonstrate how to use cluster-based permutation tests to control the family-wise error rate from multiple comparisons, and how to compute and report standard effect size metrics for cluster-based permutation tests to better convey a finding’s impact and robustness.

Moving beyond traditional approaches, “hyperscanning” provides a framework for analyzing EEG data simultaneously recorded from two or more individuals engaged in a social interaction. As noted by Kayhan et al. (2022), the traditional approach of social neuroscience involves studying one participant at a time, during passive interactions with stimuli. However, hyperscanning allows for capturing neural dynamics present across the brains of social partners engaged in dyadic (or higher order) interactions. Providing an introduction to this approach, Kayhan et al. (2022) introduce the Dual EEG Pipeline (DEEP), which is capable of performing standard preprocessing of data, as well as calculating measures of inter-brain phase alignment. An additional consideration in hyperscanning is whether to treat inter-brain dynamics as time-invariant properties that remain relatively stable throughout a given social interaction. Whereas most work to date focuses on analyzing inter-brain relations as time-invariant, Haresign et al. (2022) point out that such approaches are unable to quantify how such relations are established and maintained over time. Filling this gap, Haresign et al. (2022) provide an introduction to approaches that can be used to measure changes in inter-brain relations throughout the course of a social interaction, including analyses based on power and phase relations and Granger causality.

## 5 Conclusions and future directions

Given the untapped potential of EEG as a developmental neuroscience tool, it is our hope that researchers will increasingly apply the methods described in this special issue (and related resources) in developmental EEG studies. The articles included in this special issue all follow a similar format, including a “beginner-friendly” theoretical introduction to a given analysis technique, a step-by-step tutorial on how to apply the technique, well-commented code that can be readily utilized, and example data on which to test out the technique. The inspiration for this article format follows successful recent examples from other fields (e.g., Kievit et al., 2018; Ram and Grimm, 2009). We hope that more of such “tutorial-style articles” will be published within the field of developmental EEG, as well as cognitive neuroscience more broadly, covering a wider range of analysis approaches and techniques. At the same time, it is important to note that traditional journal articles are relatively “static” by their nature, which can impede the rapid dissemination of improvements for a given analysis approach or tutorial. To mitigate this concern, many of the articles of this special issue host their example code via an online platform that is designed with version control in mind (e.g., Github.com, OSF.io). Such platforms are particularly helpful to allow for relevant code or tutorials to be updated when bug fixes are necessary or new features are appropriate. To this end, we encourage more researchers to take advantage of such platforms when publishing a tutorial or for hosting the analysis code for an empirical article. Relatedly, dedicated tutorials take time to write, and may not be appropriate for all techniques. Thus, we additionally encourage the normalization of publishing all analysis code and data alongside empirical articles. However, going a step further, we suggest that researchers should take care to ensure such code is well-commented, easy to run across platforms, and includes additional explanations where needed to facilitate ease of understanding by both reviewers and other researchers. In essence, by publishing one’s analysis code and data, all articles can serve as “mini tutorials” for the analysis methods employed in a given study.

Looking ahead to the future, we strongly believe that EEG as a developmental neuroscience tool is well poised for a resurgence. As evidence of this, recent large-scale data collection studies have incorporated EEG as a key measure of interest, such as the Youth Of Utrecht (YOUth) Study (Onland-Moret et al., 2020), the Healthy Brain Child Development (HBCD) Study (Volkow et al., 2021), the Eurosibs Consortium (Jones et al., 2019), and the Autism Biomarkers Consortium for Clinical Trials (ABC-CT) (McPartland et al., 2020). The utility of EEG in such endeavors is not limited to the relative low-cost, ease-of-use, and unique ability to collect data across different experimental paradigms and contexts (e.g., collecting task-based EEG from awake infants in the laboratory, clinical setting, or home). As demonstrated by the articles of this special issue, EEG is an information-rich signal, capable of providing insights into developmental phenomena that cannot be captured by other methods. As the data from more developmental EEG studies (both large and small) become publicly available and organized in a standardized format, we hope that researchers will increasingly apply a diverse array of analysis techniques to maximize the utility of EEG as a developmental neuroscience tool.
